# Generation of Formates Following 20 kHz Sonication of DSPE-mPEG2000 PEGylated Phospholipid Micelles

**DOI:** 10.3390/pharmaceutics17081008

**Published:** 2025-08-01

**Authors:** Perouza Parsamian, Paul Pantano

**Affiliations:** Department of Chemistry and Biochemistry, The University of Texas at Dallas, Richardson, TX 75080-3021, USA; parsamianp@gmail.com

**Keywords:** polyethylene glycol, nanocarriers, sonolysis, nanotoxicity, ocular drug delivery

## Abstract

**Background**: Previous research has demonstrated that 20 kHz probe or 37 kHz bath sonication of poloxamers comprising polypropylene glycol (PPG) and polyethylene glycol (PEG) blocks can generate degradation byproducts that are toxic to mammalian cells and organisms. Herein, an investigation of a PEGylated phospholipid micelle was undertaken to identify low-molecular-weight sonolytic degradation byproducts that could be cytotoxic. The concern here lies with the fact that sonication is a frequently employed step in drug delivery manufacturing processes, during which PEGylated phospholipids can be subjected to shear forces and other extreme oxidative and thermal conditions. **Methods**: Control and 20 kHz-sonicated micelles of DSPE-mPEG2000 were analyzed using dynamic light scattering (DLS) and zeta potential analyses to study colloidal properties, matrix-assisted laser desorption/ionization–time of flight (MALDI-TOF) mass spectroscopy (MS) and proton nuclear magnetic resonance (^1^H-NMR) spectroscopy to study the structural integrity of DSPE-mPEG2000, and ^1^H-NMR spectroscopy and high-performance liquid chromatography (HPLC) with ultraviolet (UV) detection to quantitate the formation of low-molecular-weight degradation byproducts. **Results**: MALDI-TOF-MS analyses of 20 kHz-sonicated DSPE-mPEG2000 revealed the loss of ethylene glycol moieties in accordance with depolymerization of the PEG chain; ^1^H-NMR spectroscopy showed the presence of formate, a known oxidative/thermal degradation product of PEG; and HPLC-UV showed that the generation of formate was dependent on 20 kHz probe sonication time between 5 and 60 min. **Conclusions**: It was found that 20 kHz sonication can degrade the PEG chain of DSPE-mPEG2000, altering the micelle’s PEG corona and generating formate, a known ocular toxicant.

## 1. Introduction

**Figure 1:**
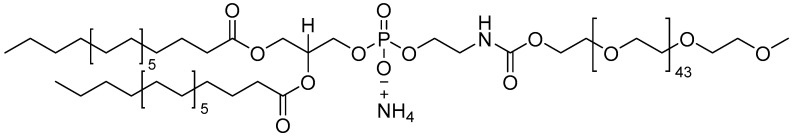
Chemical structure of the ammonium salt of DSPE-PEG2000-CH_3_ (also known as DSPE-mPEG2000).

The administration of pharmaceuticals is often challenging due to their hydrophobic properties, which make them difficult to solubilize in aqueous bodily fluids, resulting in poor adsorption and reduced therapeutic effectiveness at the target site [1,2,3,4]. In light of this, numerous polymer- and phospholipid-based nanocarriers have been developed to augment the biophysiochemical characteristics of therapeutics [3,5,6,7,8]. A key component of most of these nanocarriers is an outer coating of polyethylene glycol (PEG) that enhances nanocarrier surface properties by minimizing their aggregation and making them stealth-like, thereby enabling them to avoid an immune response [8,9]. Figure 1 shows the structure of a common PEGylated lipid, 1,2-distearoyl-sn-glycero-3-phosphoethanolamine-N-[methoxy(polyethylene glycol)-2000], commonly known as DSPE-PEG2000-CH_3_ or DSPE-mPEG2000. The DSPE-PEG family of phospholipid–polymer conjugates is amphiphilic, comprising a hydrophobic core and a hydrophilic shell; these conjugates are biocompatible and biodegradable and can be functionalized with various biomolecules for specific functions in drug delivery and imaging [10,11]. DSPE-mPEG2000 micelles are advantageous because they are generally smaller in size relative to other polymer-based drug delivery nanoparticles and they possess a low (~1 μM) critical micelle concentration (CMC), which promotes micelle stability even after injection and subsequent dilution into the bloodstream [12]. DSPE-mPEG2000 is subsequently employed to improve the biopharmaceutical properties of micelles, liposomes, and nanoparticles by increasing their colloidal stability (e.g., by minimizing aggregation) and hindering interactions between colloidal nanocarriers and blood components (e.g., decreasing the adsorption of proteins), thereby reducing uptake mediated by macrophages of the reticuloendothelial system and leading to prolonged bloodstream circulation times [8,10,13,14]. In general, tailoring of these properties can be governed by the physiochemical properties of the PEG corona that coats the nanocarrier surface including the PEG surface density, as well as the chain length, branching, and terminal groups of the PEG moiety [3,8,13,15]. Given this, it is therefore imperative that the intended architecture of the PEG corona is maintained during nanocarrier manufacturing [16].

There are numerous PEGylated phospholipid drug delivery formulations, and many strategies have been developed that involve multiple processing steps depending on nanocarrier components and/or the envisioned biomedical application [11,17,18,19,20]. Of particular concern are manufacturing processes such as sonication, homogenization, or extrusion that subject components to shear forces as part of dispersing and/or sizing operations. In particular, PEGylated lipids such as DSPE-PEG2000 and DSPE-mPEG2000 are frequently sonicated in the process of preparing of micelles and liposomes [21,22,23,24,25,26,27,28,29,30,31]; for example, the “thin-film hydration and sonication” method to prepare liposomes comprising DSPE-mPEG2000 was performed by Khutoryanskiy and co-workers using a bath sonicator [32], and by Yao, Han, and co-workers using a probe sonicator [33].

Sonication is the application of sound energy at frequencies typically above 20 kHz. Briefly, as sound waves from an acoustic transducer radiate through a solution, they cause alternating high and low pressures in the solution. During the low-pressure stage, millions of microscopic bubbles form and grow in a process called cavitation, and during the high-pressure stage, the bubbles collapse, or implode, generating extreme local conditions including high heats, pressures, and shear forces at the bubble–water interface [34,35,36,37,38]. Sonolytic frequencies typically dictate application; for example, sonochemical syntheses and the purposeful degradation of pollutants is typically performed using frequencies in the range of 350 kHz–1 MHz [38,39,40,41]; the ultrasonic-mediated release of drugs from micelles/liposomes and uptake by biological cells is generally achieved using frequencies in the range of 1–5 MHz, while non-invasive medical imaging is typically performed with ultrasound frequencies in the range of 5–15 MHz [42,43].

Herein, this investigation focuses on the lower frequency ranges of benchtop probe (20–28 kHz) and bath (37–40 kHz) sonicators, which are commonly used to clean delicate instruments and materials, disrupt biological cell membranes, break down particles into smaller sizes, and disperse and mix particles in fluids [35]. Nonetheless, even at these low frequencies, certain probe and bath sonication conditions are well-known to degrade a variety of polymers; for example, 28 kHz probe sonication of PEG-PPG di-block co-polymers [44], 20 kHz probe and 37 kHz bath sonication of PEG-PPG-PEG tri-block co-polymers [45,46], 25–28 kHz probe sonication of PEG [47,48,49], 37 kHz bath sonication of PEG and branched-chain PEGs [45,50], 40 kHz bath sonication of DSPE-PEG2000-NH_2_ [16], and 37 kHz bath sonication of DSPE-PEG2000-NH_2_ and DSPE-PEG2000-COOH [50]. In these examples, the sonolytic transformation of polymeric materials in solution can stem from (i) attacks by reactive oxygen species and free radicals, which originate from the implosive collapse of bubbles, (ii) mechanochemical effects from shear forces generated around collapsing cavitation bubbles, and (iii) pyrolysis in the hot interfacial region between bubbles and the surrounding liquid [36,38,44,49,51,52].

While it has been known for decades that the structural integrity of certain polymers can be sensitive to sonication, the demonstration that sonication of PEG-containing polymers can generate toxic byproducts is relatively recent. For example, Draper and co-workers first reported that the degradation byproducts of sonicated PEG-PPG-PEG tri-block co-polymers (also known as poloxamers, known by the trade name Pluronic^®^) can be toxic to mammalian cells and organisms [45,46]. Specifically, solutions of poloxamer 407 (i.e., Pluronic^®^ F-127) or poloxamer 188 (i.e., Pluronic^®^ F-68) at concentrations below their CMCs that were bath-sonicated for 15–240 min at 37 kHz (or probe-sonicated for 2–30 min at 20 kHz) were shown to be toxic to normal rat kidney (NRK) cells [45]; solutions of poloxamer 338 (i.e., Pluronic^®^ F-108) at concentrations below its CMC that were bath-sonicated for 15–240 min at 37 kHz were shown to be toxic to mouse macrophage RAW 264.7 cells and zebrafish embryos [46]. An equally important aspect of these works was the development and validation of a simple dialysis purification method to detoxify these samples, whereby the resultant sonicated and dialyzed poloxamer solutions had no effect on cell proliferation or zebrafish embryo viability and development. Specifically, dialysis of sonicated poloxamer solutions with membranes possessing a molecular weight cut-off (MWCO) of 300 kDa was used to render sonicated poloxamer 407 and poloxamer 188 samples non-toxic to NRK cells [45], and membranes with an MWCO of 100 kDa were used to render sonicated poloxamer 338 samples non-toxic to RAW 264.7 cells and zebrafish embryos [46].

Herein, DSPE-mPEG2000 was chosen to expand upon previous reports of the 37–40 kHz sonolytic degradation of DSPE-PEG2000-COOH and DSPE-PEG2000-NH_2_ [16,50]. Specifically, the goals of this work were to investigate the structural integrity of DSPE-mPEG2000 micelles following 20 kHz sonication and to identify any low-molecular-weight degradation byproducts that could be potentially toxic to mammalian cells. MALDI-TOF-MS analyses of 20 kHz-sonicated DSPE-mPEG2000 revealed the loss of ethylene glycol moieties in accordance with depolymerization of the PEG chain, which was congruent with indicators of micelle disruption and reduced colloidal stability of sonicated DSPE-mPEG2000 micelles as measured by ^1^H-NMR and zeta potential analyses, respectively. ^1^H-NMR spectroscopy showed the presence of formate, a known ocular toxicant, in the spectrum of 20 kHz-sonicated DSPE-mPEG2000 micelles, and HPLC-UV showed that the generation of formate was dependent on 20kHz probe sonication time between 5 and 60 min. While formates are known oxidative/thermal degradation products of PEG [38,53,54,55,56,57,58], the actual detection of formates generated by sonication of PEG or PEG-containing molecules (using probe sonicators operated in the 20–28 kHz frequency regime or bath sonicators operated in the 37–40 kHz frequency regime) has not been demonstrated. In fact, to the best of our knowledge, the only demonstration of sonolytic production of formates from a surfactant containing a short ethylene oxide chain involved 358 kHz probe sonication of Triton-X-100 [40].

## 2. Materials and Methods

### 2.1. Chemicals

1,2-distearoyl-sn-glycero-3-phosphoethanolamine-N-[methoxy(polyethylene glycol)-2000] (DSPE-mPEG2000, product No. 880120) was purchased from Avanti Polar Lipids (Alabaster, AL, USA). Deuterium oxide (D_2_O, 100.0 atom% D) and α-cyano-4-hydroxycinnamic acid were purchased from Thermo Fisher Scientific (Waltham, MA, USA). Trifluoroacetic acid (TFA, HPLC Grade), acetonitrile (ACN, HPLC Grade), sodium formate, and MALDI-TOF-MS standard (ProteoMASS™ adrenocorticotropic hormone (ACTH) fragment 18–39) were purchased from Sigma-Aldrich (Burlington, MA, USA). Polybead^®^ carboxylate microspheres (0.10 µm diameter) were purchased from Polysciences (Warrington, PA, USA). Zeta-transfer standard (product No. ZTS1240) was purchased from Malvern Panalytical (Malvern, UK). Deionized water (18.3 MΩ cm) was obtained using a Nanopure^®^ Infinity water purification system (Barnstead, Dubuque, IA, USA).

### 2.2. Preparation of DSPE-mPEG2000 Micelles

A 2.00 mM solution of DSPE-mPEG2000 was prepared by first weighing 0.0168 g (0.00600 mmol) of DSPE-mPEG2000 into a scintillation vial, followed by the slow addition of 3.00 mL of deionized water (or 3.00 mL of D_2_O for samples analyzed by NMR spectroscopy), and then stirring for 30 min at ~720 RPM.

### 2.3. Sonication of DSPE-mPEG2000 Micelles

Probe sonication was performed using a 3 mm diameter microtip sonicator probe attached to a Branson 250 Sonifier (Emerson Electric Co., St. Louis, MO, USA). An aqueous solution (1.5 mL) of DSPE-mPEG2000 was placed in a 15 mL falcon tube, which was submerged ~2.5 cm into an ice-water bath. The probe tip was centered and submerged ~0.5 cm below the surface of the DSPE-mPEG2000 solution. The sonicator was operated at 20 kHz in pulse mode (7.5-s on, 7.5-s off) at 50% amplitude, which corresponds to a power range of 30–42 W. Samples were sonicated for 5, 15, and 60 min; in the latter case, sonication was performed at 15 min intervals, and the bath’s ice-water was replaced with fresh ice-water after every interval. The mean effective calorimetric power delivered to the solution after 15 min was 14 W, as determined using the method of Hackley and co-workers [59].

Bath sonication was performed for 15 or 30 min using a Branson model 2510 bath sonicator operating at 130 W and 40 kHz. An aqueous solution (1.0 mL) of DSPE-mPEG2000 was placed in a 20 mL scintillation vial, which was positioned at the center of the bath, as detailed previously by Draper and co-workers [45]. The mean effective calorimetric power delivered to the solution after 30 min was 69 W, as determined using the method of Hackley and co-workers [59].

### 2.4. DLS and Zeta Potential

Particle size distributions and zeta potentials were determined at 25 °C using a Malvern Nano-ZS 3600 Zetasizer equipped with a 633 nm laser (Malvern Panalytical, Malvern, UK)and a fixed-angle detector position of 173°. Samples, control or sonicated DSPE-mPEG2000 solutions (both pH 6), or the calibration standard (1 mL each), were contained in a polystyrene DLS cuvette (Malvern product No. ZEN0040) or a folded capillary zeta potential cell (Malvern product No. DTS1070). For DLS analyses, three independent measurements of a sample or the Polybead^®^ standard, each comprising ten runs at 30 s/run, were acquired and averaged. For zeta potential analyses, three consecutive measurements of a sample or the zeta-transfer standard were acquired and averaged using an automated number of runs. The hydrodynamic diameters and zeta potentials of deionized water and DSPE-mPEG2000 samples were calculated using a viscosity and refractive index of 0.8872 cP and 1.330, respectively, and an absorption and refractive index of 0.010 and 1.51, respectively.

### 2.5. MALDI-TOF-MS

MALDI-TOF-MS experiments were conducted using a Shimadzu AXIMA Confidence™ MALDI-TOF mass spectrometer equipped with a 337 nm nitrogen laser (Shimadzu, Columbia, MD, USA). The matrix solution was 10 mg α-cyano-4-hydroxycinnamic acid dissolved in 1.0 mL of a solution comprising 70% ACN and 30% (vol/vol) aqueous 0.1% TFA [16,60]. In all cases, the matrix solution was mixed (2:1 vol/vol) with the ACTH standard or with a sample (a control or sonicated DSPE-mPEG2000 solution). Subsequently, 2 µL of one of these mixtures was dispensed onto different regions of a stainless-steel target plate and left to dry for at least 30 min. Instrument calibration and sample analyses were performed in positive linear mode using the following automated parameters: a power setting of 120, a profile number of 500, and 2 shots. Spectral processing was performed with Shimadzu Launchpad Software version 2.9.4 using the following parameters: a peak width of 5, peak area set to the tip of the peak, smoothing method set to average, smoothing filter width of 10, baseline filter width of 1000, threshold-apex peak detection method, and a threshold offset of 0.010.

### 2.6. ^1^H-NMR Spectroscopy

^1^H-NMR spectroscopy experiments involving control and sonicated DSPE-mPEG2000 samples were performed at 25 °C using a Bruker Avance III™ 500-MHz spectrometer (Bruker, Billerica, MA, USA). Briefly, 1 mL aliquots of a sample or a D_2_O blank was placed into a Wilmad^®^ NMR tube, and 400 scans were acquired. Data were processed through MestreNOVA Software version 16.0.0

### 2.7. HPLC-UV

Quantitative analyses were based on established HPLC-UV methodologies for the detection of formates [38,61] and were conducted using an Agilent Technologies 1260 Infinity II HPLC system equipped with a variable-wavelength detector set at 220 nm (Agilent Technologies, Inc., Santa Clara, CA, USA). A reverse-phase C18 column (Kinetex XB-C18 core–shell column, Phenomenex, Princeton, NJ, USA) was used with a mobile phase comprising 90% of a 25 mM monobasic potassium phosphate solution and 10% (vol/vol) aqueous ACN. All experiments were run for 30 min in isocratic mode at an instrument flow rate of 1.0 mL/min. Samples, control or sonicated DSPE-mPEG2000 solutions, or sonicated DSPE-mPEG2000 solutions spiked with 7.35 mM sodium formate, were prepared using an aqueous 10% (vol/vol) ACN solution. Sodium formate standards (1.0 mg/mL, 0.1 mg/mL, and 0.01 mg/mL) were prepared using the mobile phase. All sample and standard injection volumes were 20.0 µL, and the injector was rinsed with 1.0 mL of deionized water between injections.

## 3. Results

### 3.1. DLS and Zeta Potential Analyses

**Figure 2:**
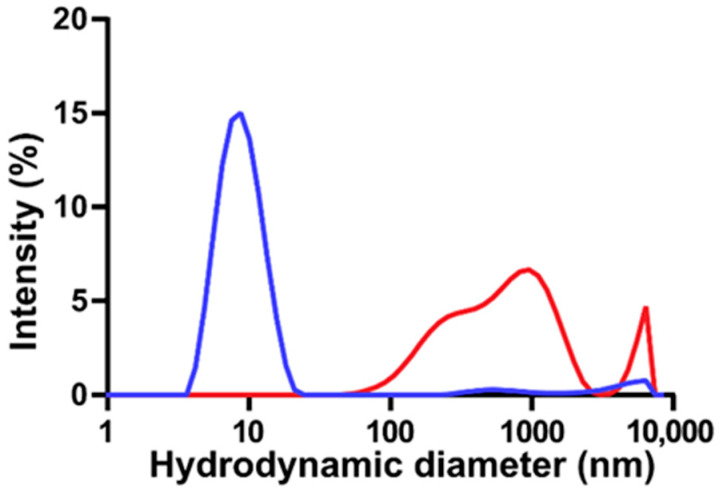
Representative DLS particle size distributions of aqueous 2.00 mM solutions of DSPE-mPEG2000 before (blue) and after (red) 1 h of 20 kHz probe sonication. Before sonication, DSPE-mPEG2000 is characterized primarily as a single major peak representing a micelle with a hydrated diameter of ~8 nm; following sonication, the micelle peak is no longer observed and a series of new structures with sizes > 90 nm appear.

The thermodynamics of DSPE-based phospholipid micelles with varying lengths of PEG appendages have been the subject of many experimental and computational investigations [12,24,50,62]. In the case of aqueous DSPE-based micelles modified with PEG2000, it has been shown that spherical micelles are formed, with an aggregation number of 76 at 25 °C, and an ~11 nm micelle comprises 20 DSPE-mPEG2000 phospholipids [12,62]. Herein, DLS and zeta potential analyses were used to study the colloidal properties of DSPE-mPEG2000 micelles subjected to sonication. Figure 2 shows a representative DLS particle size distribution of an aqueous solution of DSPE-mPEG2000 before and after 1 h of 20 kHz probe sonication. DSPE-mPEG2000 is known to spontaneously assemble in water into uniform micelles [12]. Indeed, the non-sonicated control is characterized by a single peak representing a micelle structure with an average hydrodynamic diameter of 8.2 ± 1.2 nm (*n* = 3 independent samples),which is in agreement with ~10 nm diameter sizes reported for various DSPE-PEG2000 micelles [12,24,50,62]. Following 1 h of sonication, the micelle peak was no longer observed, and only larger structures with hydrodynamic diameters > 90 nm were detected. These findings are similar to those reported by Draper and co-workers, who observed significant declines in the intensity of the DLS micelle peaks of DSPE-PEG2000-NH_2_ and DSPE-PEG2000-COOH as a function of 37 kHz bath sonication time, concomitant with the appearance of larger particles with hydrodynamic diameters > 200 nm, believed to be aggregates of hydrophobic degradation fragments [50]. The zeta potential of control DSPE-mPEG2000 micelle solutions was −30.1 ± 6.3 mV (*n* = 3), corresponding to a moderately stable colloidal system, which is to be expected since the steric stabilization provided by PEG is known to reduce the tendency of particles to aggregate [13]. Conversely, the zeta potential of 1 h probe-sonicated solutions of DSPE-mPEG2000 was less negative (−23.1 ± 5.5 mV (*n* = 3)), symptomatic of colloidal nanoparticle instability, which could be a result of the formation of aggregates of DSPE-mPEG2000 degradation fragments.

### 3.2. MALDI-TOF-MS Analyses

**Figure 3:**
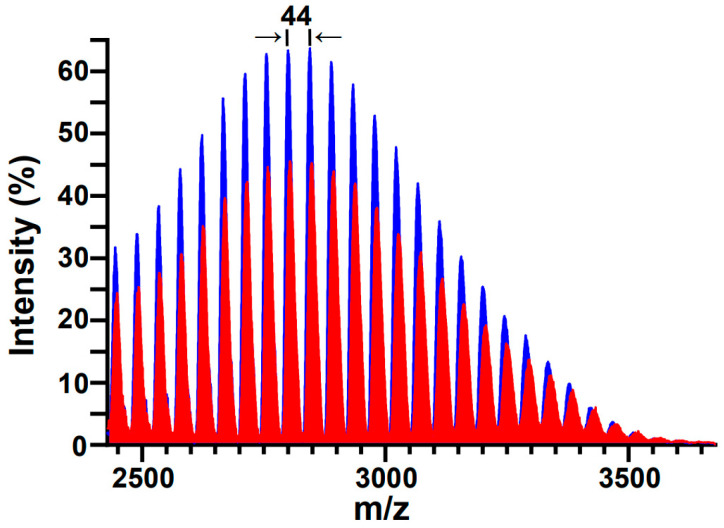
Representative MALDI-TOF-MS spectra of DSPE-mPEG2000 before (blue) and after (red) 1 h of 20 kHz probe sonication. Both spectra display the expected symmetry distinctive of a polydisperse polymer; the differences between adjacent peaks (Δ*m*) correspond to the mass unit difference (*m/z* = 44.05) between repeating units of (CH_2_-CH_2_-O)*_n_*. Post sonication, the loss of mass observed in the *m/z* range of 2428 to 3678 was 14%.

MALDI-TOF-MS has previously been demonstrated to be a useful tool in assessing the structural integrity of PEG and PEGylated molecules subjected to sonication [44,49,60]; most pertinently, Zeineldin and co-workers used MALDI-TOF-MS to demonstrate the degradation of DSPE-PEG2000-NH_2_ into smaller fragments following 40 kHz bath sonication [16]. Figure 3 shows representative MALDI-TOF-MS spectra of aqueous DSPE-mPEG2000 solutions before and after 1 h of 20 kHz probe sonication. Both spectra display the expected symmetry characteristic of a PEGylated phospholipid with a polydisperse PEG appendage, and both display the expected spacing between adjacent peaks (Δ*m*) that correspond to the mass unit difference between repeating units (*m/z* = 44.05) of ethylene glycol monomers (CH_2_–CH_2_–O)*_n_*. However, examination of the peak areas for the non-sonicated (17,896 ± 375 (*n* = 3)) and sonicated (13,755 ± 646 (*n* = 3)) DSPE-mPEG2000 samples in the *m/z* range of 2428 to 3678 reveals a 14% decrease in mass. Since the peaks in the sonicated spectrum continue to represent a homologous series of ethylene glycol monomers, the most probable sonolytic degradation mechanism involves the successive depolymerization of ethylene glycol moieties [38], in accordance with the loss of repeating units of ethylene glycol moieties observed previously in the MALDI-TOF-MS analyses of 28 kHz probe-sonicated PEG [49] and 40 kHz bath-sonicated DSPE-PEG2000-NH_2_ [16].

### 3.3. ^1^H-NMR Spectroscopic Analyses

**Figure 4:**
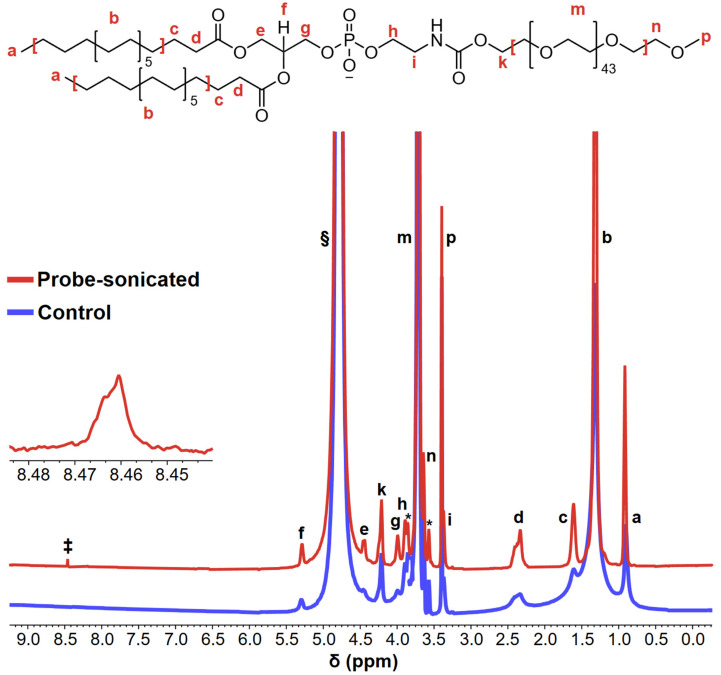
Representative ^1^H-NMR spectra of aqueous 2.00 mM solutions of DSPE-mPEG2000 before (blue) and after (red) 1 h of 20 kHz probe sonication. The lowercase letters above/adjacent to peaks correspond to the protons denoted in the DSPE-mPEG2000 structure. The peaks indicated with asterisks (*) represent J-coupling splitting between ^1^H and the natural ^13^C abundance of strong ethylene glycol units [12]; the § symbol represents the D_2_O peak; the peak denoted with the ‡ symbol at δ ~8.4 ppm represents the generation of formate in the sonicated sample. The inset represents a ~65× magnified view of the formate peak.

One of the first complete chemical shift assignments of DSPE-mPEG2000 protons was reported by Shively and co-workers [12]; these proton assignments are identified by lowercase letters adjacent to the DSPE-mPEG2000 structure shown Figure 4, which also shows the ^1^H-NMR spectra of aqueous DSPE-mPEG2000 solutions before and after 1 h of 20 kHz probe sonication. Several noteworthy differences are observed in the post-sonicated spectrum relative to the control spectrum. The first is a ~2-fold narrowing of the full width at half maximum (FWHM) of peaks “a” and “b” (i.e., the methyl- and methylene-protons of the hydrocarbon chain, respectively), and the other is a ~3-fold narrowing of the FWHM of peak “g” (i.e., the methylene protons alpha to the phosphate group). These differences are significant because appreciable narrowing of ^1^H-NMR peaks is indicative of micelle disruption [63,64,65], which is congruent with the zeta potentials recorded for sonicated DSPE-mPEG2000 samples vs. controls.

The most noteworthy result of the ^1^H-NMR experiments, as shown in Figure 4 in the spectrum of the probe-sonicated sample, is the appearance of a new peak at δ~8.4 ppm, which can be ascribed to formate HCO_2_^−^ [58,66,67]. This is a key finding because formates are known oxidative/thermal degradation products of PEG [38,53,54,55,56,57,58]; therefore, formate generation supports the sonolytic degradation of the PEG chain of DSPE-mPEG2000, congruent with the MALDI-TOF-MS results.

### 3.4. HPLC-UV Analyses

**Figure 5:**
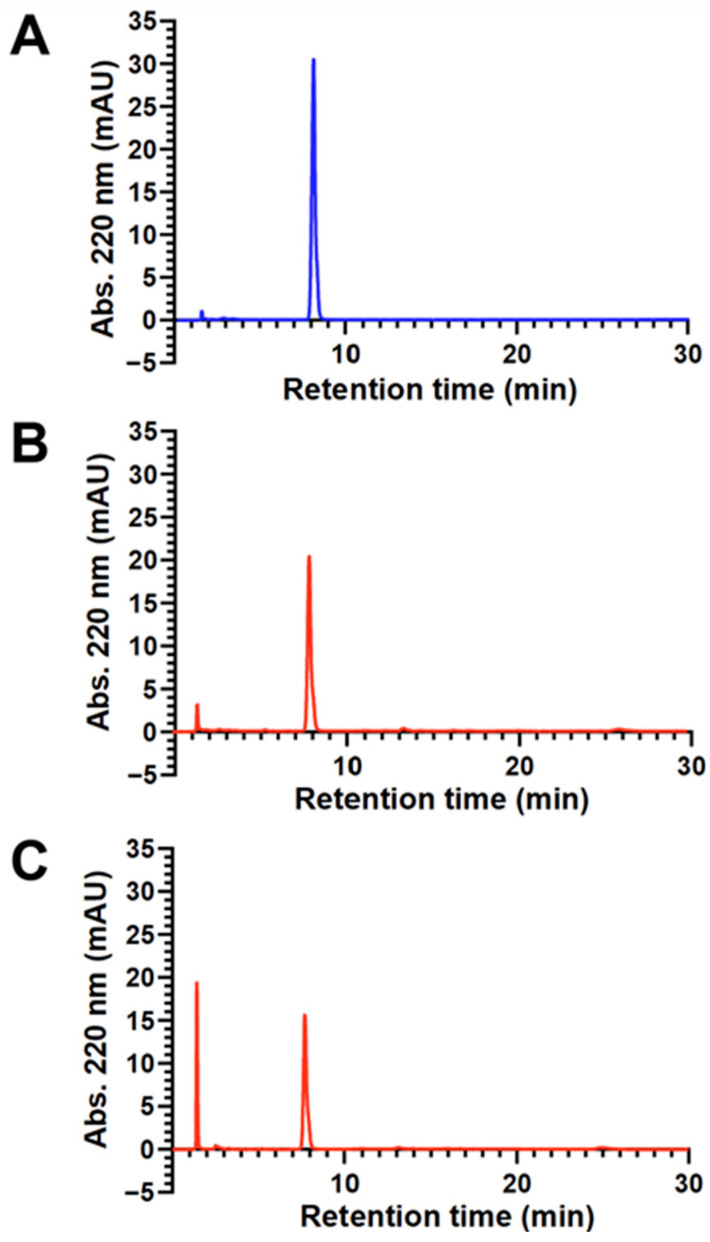
Representative HPLC-UV chromatograms (normalized to the same y-axis scale) of aqueous 2.00 mM solutions of DSPE-mPEG2000 before sonication (**A**), after 1 h of 20 kHz probe sonication (**B**), and a 1 h probe-sonicated sample spiked with 7.35 mM sodium formate (**C**). In all chromatograms, the peak with a retention time of ~8 min represents DSPE-mPEG2000, and the peak with a retention time of 1.4 min represents formate. The peak with a retention time of 1.6 min in chromatogram A represents the solvent front.

HPLC-UV analyses were employed to quantitate sonolytic formate production. Figure 5A,B show representative HPLC-UV chromatograms of DSPE-mPEG2000 before and after 1 h of 20 kHz probe sonication, respectively. The first noteworthy peak in both chromatograms appears at a retention time of ~8.0 min and corresponds to DSPE-mPEG2000. The second peak, at a retention time of 1.4 min, represents formate and appears only in the sonicated chromatogram, which also shows a corresponding decrease in the area of the DSPE-mPEG2000 peak. To verify that the 1.4 min peak was indeed formate, a sample of DSPE-mPEG2000 that was sonicated for 1 h was spiked with 7.35 mM aqueous sodium formate. As shown in Figure 5C, the only observable change in this chromatogram (up to a retention time of 30 min) was an increase in the signal intensity of the peak at 1.4 min. Next, a series of sodium formate standards between 14.7 mM and 0.147 mM were analyzed, and a calibration curve was generated where the areas of the peak at 1.4 min were found to be linear (R^2^ = 0.9999) with respect to the concentration of formate. Using this calibration curve, the concentration of formate detected in the chromatogram of the DSPE- mPEG2000 sample that was probe-sonicated for 1 h (Figure 5B) was determined to be 2.26 mM. This value is also shown in Table 1, along with those for DSPE-mPEG2000 samples that were probe-sonicated for 5 and 15 min, and the combined data indicate that formate generation is dependent on 20 kHz probe sonication time between 5 and 60 min. Table 1 also shows the results for two DSPE-mPEG2000 samples that were sonicated using a bath sonicator—the more commonly used apparatus in pharmaceutical research and development work. These results are for comparative purposes and indicate that 15 min of 40 kHz bath sonication did not produce detectable levels of formate, and that the concentration of formate detected after 30 min of bath sonication (0.25 mM) was similar to the concentration of formate detected after merely 5 min of 20 kHz probe sonication (0.29 mM).

## 4. Discussion

Herein, MALDI-TOF-MS analyses of 20 kHz-sonicated DSPE-mPEG2000 micelles revealed the loss of ethylene glycol moieties, consistent with depolymerization of the PEG chain; this finding aligned with indicators of micelle disruption and reduced colloidal stability of sonicated DSPE-mPEG2000 micelles as measured by ^1^H-NMR and zeta potential analyses, respectively. ^1^H-NMR spectroscopy of 20 kHz-sonicated DSPE-mPEG2000 micelles showed the presence of formate, a known oxidative/thermal degradation product of PEG. Formate is generated following the random scission of C-O bonds that produce end products such as ethylene glycol, which can further be oxidized to various C2 carboxylic acids and ultimately formate [38,53,54,55,56,57,58]. The presence of formate is consistent with the oxidative, thermal, and mechanochemical processes involved in the sonolytic degradation of polymers [36,38,44,49,51,52]. Detection of formates was also demonstrated using an HPLC-UV method, which additionally showed that the generation of formates was dependent on 20 kHz probe sonication time between 5 and 60 min.

There are two main implications regarding the intended biopharmaceutical performance of a DSPE-mPEG2000 micelle if the PEG chain is transformed by 20 kHz sonication. The first is the potential alteration of the micelle’s PEG corona, whose physiochemical properties are directly involved in influencing the micelle’s colloidal stability, circulation time, etc. [8,10,13,14]. The second concerns PEG degradation byproducts, such as formate, and the need to compare sonolytically generated formate levels to those reported in the literature that could elicit toxicity and/or a deleterious biological response. In the case of formate, this is a particular concern for ocular cells, such as those in the retina, optic nerve, and basal ganglia, which are quite sensitive due to their limited ability to metabolize it [68,69,70,71,72,73,74,75]. For example, an investigation by Treichel, Burke, and co-workers using cultured mouse retinal photoreceptor (661 W) cells revealed cytotoxicity after 6 h of exposure to 30 mM sodium formate [74], a concentration ~13× higher than that in the 1 h probe-sonicated DSPE-mPEG2000 samples and roughly two orders of magnitude higher than that in 30 min bath-sonicated DSPE-mPEG2000 samples (Table 1).

The ability to investigate the structural integrity of PEGylated phospholipid nanocarriers following sonication and to identify potentially toxic degradation byproducts is increasingly relevant for the ocular drug delivery field. This is because, in the case of eye drops, a drug formulation (and a potential unintended toxicant) could be administered repeatedly on a daily basis, while in the case of an intravitreal injection, the drug formulation is delivered into the vitreous humor, a space that represents a ~1000-fold smaller volume (and a ~1000-fold less dilution of potential toxicant amounts) than a formulation injected into the bloodstream. Indeed, when one considers polymer- and lipid-based colloidal nanocarrier systems that are currently being developed for ocular drug delivery applications [76,77], several approaches involve micelles or liposomes comprising DSPE-mPEG2000 that were subjected to sonication. For example, Khutoryanskiy and co-workers prepared liposomes where 30 min of sonication was employed using a bath sonicator operating at a frequency of 35–45 kHz and a power of 120 W [32], and Yao, Han and co-workers prepared liposomes where 2 min of sonication was employed using a probe sonicator operating at a frequency of 20–25 kHz and a power of 50 W [33].

It should be additionally appreciated that while these two examples represent works where the experimental details of sonication were well documented, this is not always the case. For example, a non-exhaustive survey of sixteen works since 2007 that described the preparation of DSPE-PEG2000/DSPE-mPEG2000 micelles and liposomes (and a few hybrid lipid–polymer nanoparticles or phospholipid nanoparticles containing DSPE-PEG2000 or DSPE-mPEG2000), which employed either bath sonication frequencies, powers, and times spanning 35–45 kHz, 100–120 W, and 3–30 min, respectively, or probe sonication frequencies, powers, and times spanning 20–25 kHz, 50–400 W, and 3–15 min, respectively, revealed five works that did not define the sonication power, four that did not define the sonication frequency, and seven that did not report the sonication temperature [21,22,23,24,25,26,27,28,29,30,31,32,33,78,79,80]. Furthermore, seven of the sixteen works with undefined sonication powers and frequencies, and with reported sonication times ranging from 2 to 240 min, did not even distinguish whether sonication was performed using a bath sonicator or a probe sonicator. This is important because the omission of even one critical parameter, such as sonication frequency, which is a major determinate of cavitation bubble size, makes it difficult to predict whether any sonolytic degradation of PEG could have potentially occurred, and also makes it difficult for researchers to reproduce these experiments. Coincidently, almost all of the sixteen works performed some degree of sample purification following sonication, including four works that employed dialysis that would have undoubtedly removed any low-molecular-weight sonolytic degradation byproducts (if present). Disappointingly, three of the seven works that did not distinguish whether sonication was performed using either a bath sonicator or a probe sonicator additionally did not perform any purification procedures. Finally, it should also be noted that reproducing sonication operations across different laboratories is hardly straightforward due to the spectrum of sonication devices currently available [59]. For example, with bath sonication, the age of acoustic transducers and the position of the sample in the bath are important factors in determining the effective acoustic energy delivered to a sample, while with probe sonication, the condition of the probe tip and the volume of liquid are two of the important factors [59,81].

In conclusion, despite the documentation of sonolytic degradation of PEG and PEG-containing molecules in the peer-reviewed literature, there remain works where nanocarriers containing PEGylated lipids are sonicated without evaluating the integrity of PEG or applying protocols designed to remove any sonolytic degradation products that were potentially generated. It would therefore be prudent in studies involving sonicated PEGylated phospholipid micelles and liposomes to test for the generation of low-molecular-weight sonolytic degradation products that may elicit unwanted biological responses and may indicate alteration of the intended architecture of the PEG corona.

## Figures and Tables

**Table 1:** Concentrations of formate detected in 20 kHz probe-sonicated and 40 kHz bath-sonicated DSPE-mPEG2000 solutions.

Sonication Method	Sonication Time (min)	Concentration of Formates Mean ± CI (mM)
Probe	5	0.29 ± 1.1
Probe	15	0.37 ± 0.6
Probe	60	2.26 ± 6.3
Bath	15	<LOD
Bath	30	0.25 ± 0.8

Notes: The reported concentrations of formate represent the means and 95% confidence intervals (CIs) from *n* = 2 independent samples. Briefly, concentrations of formate corresponding to the HPLC-UV peak heights observed at a retention time of 1.4 min from 20 kHz probe- or 40 kHz bath-sonicated solutions of DSPE-mPEG2000 were determined from a calibration curve generated from HPLC-UV peak heights observed at a retention time of 1.4 min from a series of sodium formate standards. The limit of detection (LOD) for the HPLC-UV method was 0.15 mM.

## Data Availability

The datasets used and/or analyzed in this work are available from the corresponding author upon reasonable request.

## Abbreviations

The following abbreviations are used in this manuscript:

PPG	polypropylene glycol
PEG	polyethylene glycol
DLS	dynamic light scattering
MALDI	matrix-assisted laser desorption/ionization
TOF	time of flight
MS	mass spectroscopy
^1^H-NMR	proton nuclear magnetic resonance
DSPE-	1,2-distearoyl-sn-glycero-3-phosphoethanolamine
mPEG	methoxy PEG
HPLC	high-performance liquid chromatography
UV	ultraviolet
CMC	critical micelle concentration
NRK	normal rat kidney
MWCO	molecular weight cut-off
D_2_O	deuterium oxide
TFA	trifluoroacetic acid
ACN	acetonitrile
ACTH	adrenocorticotropic hormone
FWHM	full width at half maximum
